# Caring potentials in the shadows of power, correction, and discipline—Forensic psychiatric care in the light of the work of Michel Foucault

**DOI:** 10.3402/qhw.v10.28703

**Published:** 2015-08-27

**Authors:** Ulrica Hörberg, Karin Dahlberg

**Affiliations:** Department of Health and Caring Sciences, Faculty of Health and Life Sciences, Linnaeus University, Växjö, Sweden

**Keywords:** Caring science, forensic psychiatric care, Foucault, philosophical examination

## Abstract

The aim of this article is to shed light on contemporary forensic psychiatric care through a philosophical examination of the empirical results from two lifeworld phenomenological studies from the perspective of patients and carers, by using the French philosopher Michel Foucault's historical–philosophical work. Both empirical studies were conducted in a forensic psychiatric setting. The essential results of the two empirical studies were reexamined in a phenomenological meaning analysis to form a new general structure in accordance with the methodological principles of Reflective Lifeworld Research. This general structure shows how the caring on the forensic psychiatric wards appears to be contradictory, in that it is characterized by an unreflective (non-)caring attitude and contributes to an inconsistent and insecure existence. The caring appears to have a corrective approach and thus lacks a clear caring structure, a basic caring approach that patients in forensic psychiatric services have a great need of. To gain a greater understanding of forensic psychiatric caring, the new empirical results were further examined in the light of Foucault's historical–philosophical work. The philosophical examination is presented in terms of the three meaning constituents: Caring as correction and discipline, The existence of power, and Structures and culture in care. The philosophical examination illustrates new meaning nuances of the corrective and disciplinary nature of forensic psychiatric care, its power, and how this is materialized in caring, and what this does to the patients. The examination reveals embedded difficulties in forensic psychiatric care and highlights a need to revisit the aim of such care.

The aim of this article is to shed light on contemporary forensic psychiatric care through a philosophical examination of results from two empirical lifeworld phenomenological studies (Hörberg, 2008; Hörberg, Sjögren, & Dahlberg, 2012) by using the French philosopher Michel Foucault's (1926–1984) historical–philosophical work (Foucault, 1988/1961, 1998/1975, 1990/1976). The studies, one from the perspective of the patients (Hörberg et al., 2012) and one from the perspective of carers (Hörberg, 2008), focus on the daily care on wards in forensic psychiatric settings. These studies describe a rigid kind of care, with few opportunities for unconditional and truly caring encounters in the forensic psychiatric settings. Instead, a vein of power permeates the care in the sense that the carers’ attitude is characterized by power, humiliation, threats, or punishment.

The essence of forensic psychiatry can be related to the assessment and treatment of people with mental disorders, which can entail an interface between two worlds that identify and regulate deviancy, that is, mental health and the law (Gordon & Lindqvist, 2007). There are different levels of security in forensic psychiatric settings (maximum, high, medium, and low), and the organization can differ between countries in terms of legislation and placement for care (Ogloff, Roesch, & Eaves, 2000; Oosterhuis & Loughnan, 2014; Salize, Dressing, & Gordon, 2007). The patients suffer from severe mental disorders and have most often committed a crime. The institutional environment is also characterized by high level of security where patients are often cared for over long periods of time (Hörberg et al., 2012). Sen, Gordon, Adshead, and Irons (2007) highlight ethical dilemmas in forensic psychiatry such as the immoderate use of segregation, the use of mechanical restraints, and physical treatment without consent.

Forensic psychiatric caring is a complex field of care due to the ambiguity of both caring for patients and applying legislation, in terms of deprivation of liberty and involuntary caring situations. For nursing staff, this entails both caring for and exerting control over the patients in this context of care. They, thus, have both a mission to provide care and to discipline (Holmes, 2005). From a nursing perspective, Jacob (2012) describes the dual role of both being “agents of care and agents of social control” and shows “that the therapeutic expertise has more to do with the humanization of the correctional structure rather than the application of a unique knowledge in the treatment of mentally ill offenders” (p. 186).

During the last two decades, several studies have focused on the complex nature of forensic psychiatric caring, the dilemma of providing care in a custodial environment, and the carers’ need to balance the dual commitments of custody and care (Burrow, 1991, 1998; Gildberg, Bradley, Fristed, & Hounsgaard, 2012; Gildberg, Elverdam, & Hounsgaard, 2010; Holmes, 2002, 2005; Hörberg, 2008; Hörberg et al., 2012; Jacob, 2012, 2014; Jacob & Foth, 2013; Maroney, 2005; Peternelj-Taylor, 1999, 2004). The research shows that the tension between care and custody in forensic psychiatric environments negatively affects carers’ possibilities to care, but that it also can be seen as a positive challenge for carers. The difficulties and ethical dilemmas that caregivers face in their caring work are highlighted (Adshead, 2000; Austin, 2001; Austin, Goble, & Kelecevic, 2009), and Austin et al. (2009) describe forensic psychiatry as a “moral minefield” due to the carers (healthcare professionals) role as “double agents” and their competing obligations.

Gildberg et al. (2012) show two forms of characteristics in staff interactions with patients in forensic psychiatric care, namely “trust and relationship-enabling care” and “behavior and perception-corrective care.” They argue that a high degree of “behavior and perception-corrective care” has a negative influence on patients and impedes the establishment of trust. Maguire, Daffern, and Martin (2014) have explored patients’ and nurses’ perspectives of limit-setting in forensic psychiatric care. Their results show that an authoritarian style characterized by controlling and indifferent behavior by nurses is experienced by patients as aggressive and disrespectful, which could result in an aggressive response toward the nurses and the limit-setting. The patients appeared to prefer nurses to have an empathic interpersonal style when setting limits, including listening and striving to understand the patient's perspective (cf. Carlsson, Dahlberg, Dahlberg, & Ekebergh, 2006). In a literature review (Gildberg et al., 2010), the interaction between staff and patients in forensic psychiatric care is characterized either by a “parentalistic and behavior-changing care” or a “relational and personal quality-dependent care.”

However, only a few studies have focused on caring in a forensic psychiatric care context from the patients’ perspective. Recent studies have featured the patients’ recovery process, where, for example, Barnao, Ward, and Casey (2015) explored patients’ perspectives on rehabilitation in forensic psychiatric care and the results showed an overall lack of person-centeredness, a varying quality of therapeutic relationships, an unclear pathway for rehabilitation, and inconsistencies in care. Olsson, Strand, and Kristiansen (2014) explored how forensic patients with a history of high risk for violence experienced the turn toward recovery. The findings are divided into three themes; a high-risk phase, a turning point phase, and attaining recovery. The transition between the phases was characterized by increased vulnerability and sensitivity. Being in a safe environment with salutary nursing was important for the patients. Tapp, Warren, Fife-Schaw, Perkins, and Moore (2013) have also shown the importance of a safe environment in the recovery process. They also highlight the importance of supportive alliances with healthcare professionals as well as peers and family. All these studies indicate the importance of good caring and a safe environment to strengthen the patients’ health processes.

## Empirical foundation

The two phenomenological empirical studies that form the basis for the general structure and the philosophical examination were carried out in a forensic psychiatric maximum secure unit in Sweden. Eleven patients, five women and six men (aged 21–42), at two different wards were interviewed in the first study. The aim was to explore and describe the meaning of patients’ experiences of their life situation at forensic psychiatric wards, with a focus on care (Hörberg et al., 2012).

Eleven carers, four women and seven men (aged 25–58), including one registered psychiatric nurse, two general nurses (RNs), and eight licensed assistant mental nurses working at seven different wards were interviewed in the second study. The aim of this study was to explore and describe the meaning of carers’ experiences of caring for patients at forensic psychiatric wards (Hörberg, 2008). Data for both empirical studies were analyzed in accordance with the Reflective Lifeworld Research (RLR) approach (Dahlberg, 2006; Dahlberg et al., 2008).

## Results from the empirical studies

The essential meanings of the results from the empirical studies are presented below. First from the patients’ perspective: “To be cared for in forensic psychiatric care” and then from carers’ perspective: “To care in forensic psychiatric care.”

### To be cared for in forensic psychiatric care

To be a patient and cared for in forensic psychiatric care entails insecurity, unreliability, and uncertainty. It also means a constant desire to want to get away from this caring, which is not perceived as care but as punishment or containment. The caring is thus experienced as being non-caring, despite the existence of “pockets” of good care.

A patient's existence in forensic psychiatric care is fragmented, without a coherent context that provides a sense of meaning to life. The non-caring care entails an isolated, unconnected, and unstable existence instead of being the protected, secure structure providing a sense of meaning. The intention of becoming free from the problems that caused the original need for the caring is overshadowed by the desire to and endeavors toward getting away from the caring. Being cared for in forensic psychiatric care entails a constant searching, without there being a definite solution or way out. The preoccupation with trying to get away from the care contributes to patients’ striving toward being as they are expected to be. The care system can make a show of a patient's right to express their opinions, but for the person being cared for it is a question of adapting to and accommodating him/herself to the persons who decide on the current regulations and routines. The patients’ daily life is characterized by fear of punishment. Feelings of fear and uncertainty are controlled via strategies aimed at holding back thoughts and feelings and just existing in the daily life on the ward, which can be quite meaningless. If the aforementioned pockets of good care are not available, the patients are left with their loneliness, which adds to their suffering. Furthermore, tensions on a relationship level grow among the patients as well as between patients and carers.

The patient's room becomes a refuge from undesired company, from the tough climate and the superficial relationships, and thus a retreat to self-chosen solitude, where one is able to feel like and be a human being. The patients long to get away from forensic psychiatric care, and they long for a dignified life with company and meaningful relationships (Hörberg et al., 2012).

### To care in forensic psychiatric care

For the carers, the caring in forensic psychiatry is made up of unreflective and contradictory correcting of patients, where the conditions are dictated by the carers and the ward culture. Correction consists of encountering patients in such a way that they gradually adapt and change. This is done by using different “corrective techniques.” The aim is to get the patients to submit to the caring regime and thus be manageable. The caring is characterized by punishment and rewards. At the same time, the caregivers want to be good examples, by showing the patients what is right or wrong in terms of speech, actions, and expression of feelings.

The carers try to break down the barriers that they perceive prevent them from engaging with the patients. The carers’ aim is to find a common arena for being with and relating to the patients. It is only when the patient has accepted and adapted to the conditions laid down by the care system that the caregivers perceive that there are possibilities for modifying the patients’ behavior.

In the daily care, the carers find themselves in situations where they are no longer acting in the professional manner that they know they should. The carers are aware of this in their own actions and/or observations of other carer's actions. They express a desire to distance themselves from the injustices against the patients, but find it difficult to act in such a manner and to stand up for this opinion, as the carers tend to be loyal to each other. The carers also sense the tensions between the patients. They are aware that the confines of the environment on the ward, its culture, and structure influence everyone there.

Being carers, they know that they have the power to influence the conditions for the patients’ care. At the same time, the carers feel that they are powerless against the patients who do not allow themselves to be submitted to the corrective measures that occur and to the “modification” of their behavior. As a consequence, feelings of hopelessness and indifference can be generated in the carer (Hörberg, 2008).

## Developing a general structure

The results from the two empirical studies raised new questions about how forensic psychiatric caring is formed. We thus wanted to explore them and the meanings they convey once more. The essential meanings of the two studies were analyzed together in a new phenomenological meaning analysis to form a general structure in accordance with the methodological principles of RLR (Dahlberg et al., 2008). Such an analysis can be seen as an integrating synthesis and abstraction of the first-level results, now integrating both patients’ and carers’ experiential perspectives. Based on the questions, the phenomenon of this new analysis was “caring in forensic psychiatric care.” Secondly, a philosophical examination was conducted to further examine the understanding of the phenomenon. Dahlberg et al. (2008) clarify that external materials such as theories or philosophy should not be included in the analysis of empirical data in phenomenological studies because of the risk that strong theories may silence the soft voice of the lifeworld. RLR advises researchers instead to further examine their data, with the support of theories and philosophy, after the empirical analyses are completed. The lifeworld data are stronger with such an approach and can contribute in a creative way to gain a more thorough understanding of the phenomenon.

## The new analysis

The new analysis for a general structure started with an open reading of the essential meanings of the two empirical results, guided by questions such as “What characterizes forensic psychiatric caring?” “How can the forensic psychiatric caring be understood as a caring practice?” In accordance with RLR, the analysis can be described as a movement between whole-parts-whole and in terms of “figure and background” (Dahlberg et al., 2008). Patterns of meanings from one of the studies worked as a figure against patterns of meanings from the other study, and vice versa. Every conceivable pattern of meanings was examined in relation to each other in different combinations in the search for new and more abstract structures of meanings. The analysis process indicated openness and sensitivity toward the phenomenon in focus and to the initial lifeworld experiences, and the researchers adopted a bridling attitude in their process of understanding (Dahlberg et al., 2008). Finally, a general structure emerged, illuminating meanings of forensic psychiatric caring, based on the experiences of both the patients and the carers.

The next stage was to further the analysis with the support of the French philosopher Michel Foucault's (1926–1984) historical–philosophical work, *Madness and Civilization: A history of insanity in the age of reason* (1988), *Discipline and punish: The birth of the prison* (1998), *The history of sexuality. Vol. 1. The will to knowledge* (1990). The choice of Foucault's texts was made based on how the results displayed similarities with historical descriptions of institutions for mentally ill persons and prisoners. In the philosophical examination, we let selected parts of Foucault's texts to shed light on the essential meanings that formed the general structure. As in the previous analyses, this process can be understood in terms of “figure and background” where meanings were understood in relation to each other.

## Results

First, the general structure of the meanings from the results of the two empirical studies is described, followed by the philosophical examination, presented in terms of the three constituents: *Caring as correction and discipline*, *The existence of power*, and *Structures and culture in care*.

### General structure

The caring at the investigated forensic psychiatric wards appears to be contradictory and is characterized by an unreflective (non-)caring attitude, which opens up for an inconsistent and insecure existence for both patients and carers. A social and existential game, where freedom is in focus, is being acted out in the daily life on forensic psychiatric wards. True caring is difficult to discern and grasp in this context because the prevalent caring culture and the care system's structure form a hinder for such caring. It is instead characterized by a movement back and forth between power and lacking power and between struggle and resignation. Different means are used for exercising power, for coping with the struggle, and counteracting a sense of resignation. The most prominent feature in the caring is the desire for modifying behavior through punishment and rewards. The “caring” tools that are available are correcting structures, power, and seclusion.

### Caring as correction and discipline

The empirical results show that carers encounter the patients with a correction style characterized by strictness, discipline, and power. They have a guarding attitude focusing on maintaining structure and order. The patients have to submit to the conditions of care and recognize what the carers want from them and how the carers want them to behave. It is a question of adapting and obeying instructions on how to behave in order for the superficial caring to lead in the direction where the light of freedom can be seen. When Foucault (1998) describes the emergence of prisons at the turn of the 18th and 19th centuries, he illustrates how the focus on punishment changed to include correction, with the purpose of not only punishing the body. However, the changes were on a theoretical level and punishment was retained, concealed under the concept of correction, and he describes this as a moral way to accept prisons. He also describes how the body was used as a tool to punish the individual by deprivation of liberty, which illustrates how the punishment in a profound way affected everyone, that is, the aim was to “reach the soul.” Our studies show how patients perceive the deprivation of liberty as a punishment with feelings of uncertainty. This could be seen in contrast to the carers who perceive the deprivation of liberty as caring for the patients. Foucault (1998) states that the punishment and manipulation of the body were made from a distance, “in the proper way, according to strict rules, and with a much ‘higher’ aim” (p. 11) and that the simple form of “deprivation of liberty” is the character of the prison. The idea was that the punishment and the crime should be closely linked to each other. Foucault says: “To find the suitable punishment for a crime is to find the disadvantage whose idea is such that it robs forever the idea of a crime of any attraction” (Foucault, 1998, p. 104). It has a discouraging effect on patients who internalize the surveillance by being accommodating. The patients also develop different strategies to outwit the surveillance by the staff in situations when they are visible, that is, the patients learn to demonstrate results in terms of submission and adaptation.

The empirical results further illustrate how the patients feel lonely and how they long for a dignified life with companionship and meaningful relationships. This could be understood in relation to Foucault (1998) who describes how prisoners are strategically subjected to solitude. He says, “Placed alone in the presence of his crime, he learns to hate it, and, if his soul is not yet blunted by evil, it is in isolation that remorse will come to assail him” (Foucault here refers to Beaumony and Tocqueville, p. 237). This statement highlights the meaning of how the carers “wait out” patients by leaving them in solitude and how they describe their caring role to correct patients’ behavior. Foucault (1988) describes that, “The therapeutics of madness did not function in the hospital, whose chief concern was to sever or to ‘correct’” (p. 159).

Furthermore, Foucault (1998) explains how the body was discovered as an object during the classical age. The power was now concentrated on the body that could be “manipulated, shaped, trained, which obeys and responds” (p. 136). He also highlights that the focus was on the notion of “docility,” which provides the link between the analyzable and the manipulable body. “A body is docile that may be subjected, used, transformed and improved” (p. 136). These ideas link with the empirical results, which show that patients are forced to comply with the regulations and that carers use methods to make patients docile. This can be seen in the carers’ narratives about how they care for patients and at the same time showing that they focus on what they perceive is possible to modify.

In summary, this part of the examination shows that correction is profoundly based in forensic psychiatric caring and forms the foundation for caring. The care that originates in correction, behavior modification, and discipline generates consequences both for those who are exposed to this care and those who deliver it.

### The existence of power

The empirical results show how the forensic psychiatric caring is permeated by power and powerlessness, which act together and are visible among both carers and patients. The corrective structure, with disciplinary actions, favors the exercise of power and at the same time undermines the possibility for true caring. The power balance between carers and patients could be described as a game, with one stronger and one weaker party. Foucault (1990) describes the meaning of power and points out the importance of existing force relations that are to be found in the context of power. He says:

It seems to me that power must be understood in the first instance as the multiplicity of force relations immanent in the sphere in which they operate and which constitute their own organization … (p. 92)

According to Foucault (1990) a power relation can never be static; instead, it operates continuously in a complex movement. He points out that power is everywhere, because it comes from everywhere in various forms. He also expresses that, where there is power, there is resistance and that resistances are “as an irreducible opposite.” (p. 96). Thus, resistances are where the power is, and the resistors cannot be in an outside position in relation to power. Instead, the power and the resistances presuppose each other. Foucault emphasizes that there is no given place for these resistances, which instead are everywhere in the power network. Foucault's description of resistance in power relations can be understood and at the same time illustrate the meaning of the patients’ behavior as a counterbalance in an unequal force relation. However, carers also feel powerlessness in relation to patients who, for example, do not respond to the carer's offers or demands. The carers and patients are also divided into groupings, and there are barriers between them that are maintained by both parties. There is a certain loyalty within the groupings, more based on sharing common conditions than on any deeper level of kinship, but they are dependent on each other within the group and can make use of and protect each other. The patients stick together superficially; they become stronger together and thus can cope longer. Foucault's analysis of power relations can help us understand this complexity of power.

The carers appear to be satisfied when the patients adjust to their requirements. Foucault (1990) points out that power relations “have a directly productive role, wherever they come into play” (p. 94). Power could be seen as a tool for the carers to reach the overall purpose for the forensic psychiatric care, that is, that the patients reintegrate into society. However, it is not certain that a patient has given up the struggle against the carer. Instead, the patient may have changed his/her strategies to maintain their balance. The empirical results show how power games between carers and patients appear to be predominant in the everyday life on the ward.

Based on Foucault's view on power relations, we could understand the meaning of the forensic psychiatric care where carers and patients try to cheat each other. In one way, both parties are winners and at the same time both are losers in relation to the meaning of true caring. Because of the inequality in the power relation, the patients are forced to lose the struggle against the carers and adapt to existing rules. Foucault's philosophy shows how difficult it is to discern and grasp the movement and manifestations of power. The empirical results show that the desire for freedom drives patients to cope with and to endure the care given. Power games, thus, provide possibilities for the patients to reach their goal, that is, freedom, and to help them to cope with the (non-)caring that is characterized by discipline, correction, and power.

In summary, the existence of power relationships appears to be unavoidable where discipline and the relative strength of the two parties are imbalanced. This part of the examination shows that that power can manifest itself in many ways. The examination reveals the importance of the power game and its influence on what could be caring in forensic psychiatric care. The patients cannot get any rest and peace in the caring, they are instead always struggling against something or for something else.

### Structures and culture in care

The empirical results show that the existing surveillance makes it difficult for patients to relax and to be spontaneous. Foucault (1998) expresses that institutions like prisons consist of a variety of instruments for subjugating inmates, and at the same time making the latter useful by processing them in a deliberate way. Furthermore, he points out that the “prison network” “with its systems of insertion, distribution, surveillance, and observation has been the greatest support, in modern society, of the normalizing power” (p. 304). Surveillance is thus an essential part of the power structures that are incorporated in the institutions. Foucault (1998) clarifies that the disciplinary power is exercised through its invisibility and at the same time it forces the inmates to always be visible. “It is the fact of being constantly seen, of being able always to be seen, that maintains the disciplined individual in his subjection” (p. 187). The empirical results show what it is like for patients to feel monitored and how they want to escape this, for example, by staying in their rooms. The patient's own room is seen as conditional freedom.

In the same way that patients have internalized the surveillance, they also know that they have to behave in all situations where they are visible for it. Foucault (1998) describes this as “the soul is the prison of the body” (p. 30) and further as “A ‘soul’ inhabits him and brings him to existence, which is itself a factor in the mastery that power exercises over the body” (p. 30). The quotes illustrate the meaning for patients in having a safe haven in one's own room, which offers a rest from surveillance, and also how patients quickly learn the conditions of the care system and adapt themselves to prevailing rules in the care setting in favor of benefits and privileges. They also bide their time, forgetting their situation for the moment.

Foucault (1998) describes the principle of panopticon for surveillance like “a machine for creating and sustaining a power relation independent of the person who exercises it” with the aim that “the inmates should be caught up in a power situation of which they are themselves the bearers” (p. 201). Foucault contrasts this to the principle of the dungeon, which entails darkness and seclusion. He argues that full lighting traps the inmate/patient better than the darkness and states that “Visibility is a trap” (p. 200). A panopticon can be applied in many different ways in different contexts.

It is a type of location of bodies in space, of distribution of individuals in relation to one another, of hierarchical organization, of disposition of centres and channels of power, of definition of the instruments and modes of intervention of power …. (Foucault, 1998, p. 205)

The empirical results show that there are interactions between the carers’ all-seeing and the patients’ feelings of always being visible. According to Foucault (1998), there are consequences with the surveillance for the monitored patients because they are forced to behave in a certain way. Each individual patient has to discover for him/herself that in the long run it is a question of giving up the struggle against the carers’ conditions to have the chance of “becoming free.” They thus have to lose the struggle. Foucault expresses that, “it gives ‘power of mind over mind’” (p. 206). When we look at how patients strategically show favorable behavior for carers, especially in situations where they are “visible,” it is clear how, as a consequence, their real health problems are overshadowed.

In summary, this part of the examination shows that traditional ways of coping with and supervising individuals who have been deprived of their liberties remain but appear in a new form. The institution's structure provides the opportunity for surveillance of the patient in forensic psychiatric care through its all-seeing, and the patients can either avoid this or utilize it by presenting themselves in a “pliable” way.

## Conclusive reflections

The philosophical examination of the empirical results (in the general structure) illustrates new meaning nuances of the corrective and disciplinary nature of forensic psychiatric care, its power and how this is materialized in caring, and what this does to the patients. The examination makes embedded difficulties visible in forensic psychiatric care and highlights a need to revisit the intention of such care.

Care, in general, aims to support patients and their health processes, so that they can heal from illness and learn how to develop well-being, even if illness is still there (Dahlberg, Todres, & Galvin, 2009; Todres, Galvin, & Dahlberg, 2007, 2014). However, the empirical studies together with the philosophical examination show that this is not the predominant concept in the investigated forensic psychiatric care unit. Our intention is not to generalize the findings to be valid in all forensic psychiatric settings. We want instead to highlight the complexity in this special kind of care due to the dual task of, on the one hand, caring for patients with complex psychiatric illness and, on the other hand, preventing new crimes and minimizing acts of violence as a protection for society. Supported by our philosophical analysis of the empirical results, we maintain that there are built-in risks in forensic psychiatric care, that the caring potentials become overshadowed by ideas of correction and discipline. Several studies, both explicitly and implicitly, focus on this problem (cf. Gildberg et al., 2010, 2012; Holmes, 2002, 2005; Jacob, 2012, 2014; Jacob & Foth, 2013). In such prevailing circumstances, there needs to be a change in favor of more genuine, health supporting caring. In particular, the care must be developed in such a way that the patients do not see any need for making up to the staff, but sincerely desire to focus on living a healthy life and abstaining from committing crimes.

Ideas from person-centered care (Ekman, 2014) and lifeworld-oriented care (Dahlberg et al., 2009; Dahlberg & Segesten, 2010; Todres et al., 2007, 2014) are being vociferously propagated for. The essential meaning of such care is that it is health-oriented and aims at patient participation in both health and caring processes. Contrary to the forensic psychiatric care that we have examined, such care emphasizes the need to see the individual, his/her resources as well as his/her existential and daily network. There is also a fundamental desire to get in touch with the human being who is a patient, getting to know what is meaningful and vital to him/her to develop the best care. In such care power or adaptational games do not suit, at least not if the intention is to move the patients toward a position characterized by health, which will prevent them from new crimes or other violent situations that are usually the cause of the need for forensic psychiatric care. Furthermore, the problem does not concern whether there should be boundaries or not. Eriksson and Wiklund Gustin (2014) describe the meaning of health and care in a monastic environment for persons with mental health problems. They show how the monastery is a place characterized by possibilities for simultaneously providing freedom within boundaries, calm and intensity, privacy and relations, demands and confirmation. A major principle is the contemplation of the human being, which makes a difference to the person's sense of dignity. There is no dualism here, instead the essence of caring means seeing the possibilities for existence, which is characterized by complexities, dualities, and in-betweens (Dahlberg, 2013). Such a context displays a number of caring qualities that are missing in many forms of care, not the least in forensic psychiatric care.

All person-centered care (Ekman, 2014) and lifeworld-oriented care (Dahlberg et al., 2009; Dahlberg & Segesten, 2010; Todres et al., 2007, 2014) is characterized by “patient participation.” Ashworth, Longmate, and Morrison (1992) argue that true participation needs to “be grasped if the nursing and other health professions are to substantiate any ethical claims. These professions are widely understood to have human interpersonal relationships at heart. To be insufficiently attentive to what have been shown to be the requirements of participation places the nurse or other health care professional in danger of treating the patient as less than a proper human being” (p. 1438). The researchers emphasize that patients “may flounder in circumstances” of care ruled by taken-for-granted assumptions” that make them “unable to feel that their contributions will be received as worthy.” Our analysis concurs with Ashworth et al. in that in such care the patients’ “sense of identity and self-esteem may, at every moment, be under threat” (p. 1438).

Furthermore, in supporting patients’ health processes, carers need to confirm and be in contact with the patients’ suffering. Vincze, Fredriksson, and Wiklund Gustin (2015) explore how nurses working in forensic psychiatric care understand and approach patients’ experiences of suffering in different ways: by ignoring suffering, explaining forensic care as a cause of suffering, ascribing meaning to suffering, or by being present in suffering. The authors clarify that being present in the encounter with suffering patients is a real challenge for nurses, both in relation to the patients’ afflictions and to their own reactions. The carers need courage to stay with the patients, reflecting together with them on the meaning of their suffering.

Hörberg (2014) describes how caring science based on a lifeworld approach can provide a theoretical foundation in the development of forensic psychiatric caring. The following areas are highlighted as being significant; the need for a patient perspective and a caring attitude, and the necessity of focusing on health and learning instead of being corrected. Based on the above, we maintain that forensic psychiatric caring needs to be questioned as well as challenged by scientifically founded knowledge of what true caring means. We also argue that such a caring science perspective can contribute to the promotion of patients’ health processes without jeopardizing the safety of the patients or the staff—more likely the opposite.

Finally, there are reasons to believe that counterproductive patterns of power, discipline, and correction also exist in psychiatric or mental health care in general. This care has traditionally included behavioristic and other therapies that do not pay attention to the patient's lifeworld. As Carlsson et al. (2006) and Lindwall, Boussaid, Kulzer, and Wigerblad (2012) show the meaning of caring that includes attendant carers, who allow themselves to be touched by patients’ stories and acting on well-expressed as well as unspoken messages. In conclusion, the value of person-centered and lifeworld-oriented care must be further evaluated and then established in all forms of psychiatric care.
